# Foreign-born health workers in Australia: an analysis of census data

**DOI:** 10.1186/1478-4491-11-69

**Published:** 2013-12-31

**Authors:** Joel Negin, Aneuryn Rozea, Ben Cloyd, Alexandra LC Martiniuk

**Affiliations:** 1 Sydney School of Public Health, University of Sydney, Sydney, Australia; 2 University of Nebraska Medical Center, Omaha, NE, USA; 3 George Institute for Global Health, Sydney, Australia; 4 Dalla Lana School of Public Health and Bloomberg School of Nursing, University of Toronto, Toronto, Canada

**Keywords:** Australia, Doctors, Health workforce, Migration, Nurses

## Abstract

**Background:**

Provide an up-to-date national picture of the medical, midwifery and nursing workforce distribution in Australia with a focus on overseas immigration and on production sustainability challenges.

**Methods:**

Using 2006 and 2011 Australian census data, analysis was conducted on medical practitioners (doctors) and on midwifery and nursing professionals.

**Results:**

Of the 70,231 medical practitioners in Australia in 2011, 32,919 (47.3%) were Australian-born, with the next largest groups bring born in South Asia and Southeast Asia. In 2006, 51.9% of medical practitioners were born in Australia. Of the 239,924 midwifery and nursing professionals in Australia, 127,911 (66.8%) were born in Australia, with the next largest groups being born in the United Kingdom and Ireland and in Southeast Asia. In 2006, 69.8% of midwifery and nursing professionals were born in Australia. Western Australia has the highest percentage of foreign-born health workers. There is a higher percentage of Australia-born health workers in rural areas than in urban areas (82% of midwifery and nursing professional in rural areas are Australian-born versus 59% in urban areas). Of the 15,168 additional medical practitioners in Australia between the 2006 and 2011 censuses, 10,452 (68.9%) were foreign-born, including large increases from such countries as India, Nepal, Philippines, and Zimbabwe. We estimate that Australia has saved US$1.7 billion in medical education costs through the arrival of foreign-born medical practitioners over the past five years.

**Conclusions:**

The Australian health system is increasingly reliant on foreign-born health workers. This raises questions of medical education sustainability in Australia and on Australia’s recruitment from countries facing critical shortages of health workers.

## Background

The World Health Organization (WHO) estimates that there is a global shortfall of more than 2 million doctors, nurses and midwives to meet the minimum recommended density [1]. This shortage of human resources for health negatively impacts health outcomes [2] with local shortages worsened by the migration of health workers from low- and middle-income countries (LMICs) to high-income countries [3].

The WHO World Health Report of 2006 calculated that 25% of doctors and 5% of nurses trained in African countries were working in high-income countries [1]. For instance, in the United States of America, 25% of physicians trained overseas – 64% of them in LMICs – as well as 4% of nurses [4]. High-income countries often actively recruit overseas-trained health workers, with a study in the United Kingdom finding that 41% of overseas-trained nurses had migrated due to active recruitment [5].

Even though health worker migration to high-income countries is occurring, countries such as Australia continue to face persistent shortages, particularly in rural and outer-metropolitan areas [6]; a situation compounded by trends towards health workers’ decreased work-hours, increased demand due to an ageing population [7] and an ageing health workforce [8]. Health Workforce Australia, a government agency tasked with coordinating the national health workforce, estimates that by 2025, there will be a shortage of 109,000 nurses and 2700 doctors along with mal-distribution across the country [9]. Australia has expanded the number of entry places for medical education but there are limitations on the availability of internships, leading some students to complete coursework requirements successfully but not the clinical requirements needed to progress to full medical registration [10].

One of the solutions to which Australia and other high-income countries have resorted in order to address these production and distribution shortages is a reliance on and recruitment of international medical graduates [11]. In the late 1990s, Australia introduced policies to encourage international medical graduates to work in Australia. The Department of Health and Ageing (DoHA) directed funding to rural workforce agencies and legislation to encourage foreign graduates to work in rural Australia. Overseas-trained doctors are only able to access national insurance scheme benefits if they practise in a defined “District of Workforce Shortage”. Partly as a result, the DoHA has estimated that international medical graduates comprise approximately 39% of the medical workforce in Australia and 46% of general practitioners in rural and remote locations [6]. This situation is forecast to continue, with the Health Workforce Australia report noting “continued reliance on poorly co-ordinated skilled migration to meet essential workforce requirements – with Australia having a high level of dependence on internationally recruited health professionals” [9]. This despite production self-sufficiency being established as a formal goal in the National Health Workforce Strategic Framework in 2004 [12].

To address some of the concerns of “brain drain” from LMICs, the Commonwealth Code of Practice for the International Recruitment of Health Workers was adopted by Commonwealth Health Ministers in 2003. This serves as a “framework within which international recruitment should take place” and is “intended to discourage the targeted recruitment of health workers from countries which are themselves experiencing shortages” [13]. The code also suggests that high-income countries consider how to recompense LMICs for the recruitment of their health workers.

Given the complexity of this issue and the importance of adequate and appropriate human resource management for health and budgetary reasons, it is necessary to understand the magnitude and trends of health workforce distribution data in Australia, with a particular focus on overseas migration. For the past few years, the Australian Institute of Health and Welfare (AIHW) has conducted surveys of doctors and nurses in Australia and published information on the national health workforce. The AIHW data, however, are limited. The 2010 national survey, for example, excluded Queensland and Western Australia from their data collection [14]. Furthermore, the 2010 survey did not collect data on country of medical qualification and while the new Medical Board of Australia data includes such information, as of late 2012 that information is not yet available. The recent parliamentary report on overseas-trained doctors states that there are “substantial gaps and inconsistencies in national medical workforce data” [6].

To be able to address Australian health workforce sustainability challenges, more data is needed on health worker production and migration. Additionally, the WHO Global Code of Practice on the International Recruitment of Health Personnel recommends that countries report data on the migration of health staff and establish research programmes on migration [15]. We therefore aim to address some of the shortcomings in knowledge of international health worker distribution and migration in Australia by using the 2011 and 2006 Australian census data to provide an up-to-date national picture of the medical, midwifery and nursing workforce in Australia, focusing on those who are foreign-born.

## Methods

This study uses data from the 2011 Australian census [16]. Details on the methods used by the Australian census in 2011 are available online [17]. In brief, by law, the census is conducted every five years. Data collection is conducted mainly on foot by approximately 43,000 collection staff. Everyone in Australia is legally required to complete a census form. The census includes all people in Australia on the census night, which, for the 2011 census, was 9 August.

The census provides information on everyone in Australia rather than being a survey of sampled respondents. Furthermore, the census uses consistent measures across years so that comparison with the 2006 census is possible and thus trends in health worker migration can be examined. Limited 2001 Australian census data is available from Organisation for Economic Co-operation and Development (OECD) reports and is used for some analyses [18].

The analysis in this paper focuses on those who self-report their occupation as “medical practitioner” or “midwifery and nursing professional”. Occupation is based on the main job held during the week prior to census night. According to census definitions, medical practitioners include general practitioners, specialist physicians, surgeons, psychiatrists and others who would be classified as practising medical doctors. The midwifery and nursing professional category includes midwives, registered nurses and nurse managers; it excludes enrolled nurses. Individual respondents defined themselves based on whatever occupation was most appropriate in response to the question of their current occupation. So individuals who trained as nurses but no longer work as nurses will not be counted as nurses. The census also captures information on state of work, rural or urban residence, country of birth, and year of arrival in Australia for those born overseas. Year-of-arrival data are used to examine those who arrived in Australia from January 2001 to August 2011.

While acknowledging that country of birth is not a perfect measure of an individual’s country of origin, it provides useful information for this study on health worker workforce and migration. The most likely alternative measure relevant to health workers – country of qualification – is problematic for a number of LMICs in the Pacific region [19] and in Africa [20], which do not have sufficient training facilities for health workers or, in some cases, have no training facilities. Therefore prospective health workers in these countries have no choice but to train in other countries. For example, a number of Pacific nationals are currently undergoing medical education in Cuba due to limited opportunities in their own countries [21]. Therefore, an indicator of medical practitioner emigration based only on country of qualification would incorrectly state that a quarter of sub-Saharan African countries and the majority of Pacific Island countries would have lost zero physicians to emigration. Furthermore, in situations where individuals train in a third country, such as that of Pacific nationals in Cuba, using country of qualification would attribute the “brain drain” to Cuba rather than to the Pacific Island country. It is acknowledged that some people in the dataset will have migrated to Australia as children and undertaken their training in Australia yet will be classified as overseas-born. While acknowledging that issues of identity are necessarily complex, covering location of residence, ancestry, citizenship, employment and ethnicity [22], using country of birth in the absence of more detailed data allows consistency across countries and across years.

Authors had full access to the data used in this study. Analysis was conducted using Microsoft Excel. An ethical review was not required for this study, as anonymised pre-collected publicly available data were used.

## Results

As of 2011, there were a total of 70,231 medical practitioners and 239,294 midwifery and nursing professionals in Australia. Of the medical practitioners for whom country of birth is known, 32,919 (47.3%) were Australian-born, with the next largest percentage being born in South Asia (11.7%) and Southeast Asia (9.4%) (Table 1). Among midwives and nurses, 157,911 (66.8%) were Australian-born, followed by those born in the United Kingdom and Ireland (9.8%) and Southeast Asia (5.6%).

**Table 1:** Medical practitioners and midwifery and nursing professionals by country or region of birth, 2011

**Country or region of birth**	**Medical practitioners**	**Percentage**	**Midwifery and nursing professionals**	**Percentage**
Australia	32,919	47.3%	157,911	66.8%
South Asia	8,155	11.7%	8,339	3.5%
Southeast Asia	6,528	9.4%	13,291	5.6%
United Kingdom and Ireland	6,328	9.1%	23,052	9.8%
Sub-Saharan Africa	3,211	4.6%	6,719	2.8%
Northeast Asia	2,988	4.3%	6,534	2.8%
North Africa and Middle East	2,655	3.8%	1,098	0.5%
Southeast and Eastern Europe	1,785	2.6%	2,540	1.1%
Western and Southern Europe	1,593	2.3%	3,773	1.6%
New Zealand	1,529	2.2%	7,335	3.1%
North America	923	1.3%	1,491	0.6%
Pacific, including Papua New Guinea	519	0.7%	2,952	1.2%
South and Central America and the Caribbean	413	0.6%	1,266	0.5%
Central Asia	120	0.2%	98	0.0%
Total with valid data	69,666	99.2%	236,399	98.8%
Not stated or inadequately described	565	0.8%	2,895	1.2%
Total	70,231		239,294	

The state or territory with the highest percentage of overseas-born medical practitioners is Western Australia, with 61.3%, while Tasmania has the lowest percentage, at 48.1% (Table 2). There are relatively high percentages in Western Australia of medical practitioners born in the United Kingdom and, Ireland and in sub-Saharan Africa while doctors born in South Asia represent a high percentage in the Northern and Australian Capital Territories.

**Table 2:** Country or region of birth (selected) of medical practitioners by state or territory, 2011 (excluding those for whom country of birth is unknown)

**Country or region of birth**	**Australian Capital Territory**	**New South Wales**	**Northern Territory**	**Queensland**	**South Australia**	**Tasmania**	**Victoria**	**Western Australia**
Australia	48.7%	45.3%	48.1%	50.9%	49.3%	51.9%	49.4%	38.7%
Total born overseas	51.3%	54.7%	51.9%	49.1%	50.7%	48.1%	50.6%	61.3%
South Asia	15.4%	12.6%	13.1%	11.1%	11.9%	12.8%	11.2%	9.9%
Southeast Asia	8.7%	9.2%	8.3%	6.4%	12.8%	3.8%	10.4%	12.2%
United Kingdom and Ireland	8.8%	7.7%	8.8%	10.0%	8.7%	11.6%	7.4%	16.0%
Northeast Asia	3.9%	6.4%	1.6%	2.7%	2.4%	1.2%	4.5%	2.3%
North Africa and the Middle East	2.0%	4.9%	1.2%	2.9%	2.5%	3.4%	4.4%	2.5%
Sub-Saharan Africa	4.1%	3.9%	6.5%	5.7%	3.3%	5.6%	3.1%	9.1%
New Zealand	2.6%	1.9%	3.2%	2.9%	1.7%	2.1%	1.9%	2.5%
Other countries	5.8%	8.2%	9.1%	7.5%	7.4%	7.5%	7.6%	6.8%

Among midwifery and nursing professionals, the highest percentage of overseas-born professionals is also in Western Australia (48.3%) (Table 3). Almost 23% of the nursing and midwifery workers in Western Australia were born in the United Kingdom and Ireland. Western Australia is also the state with the highest percentage of nurses and midwives born in sub-Saharan Africa. The highest percentage of nurses born in Southeast Asian countries is in New South Wales and Victoria.

**Table 3:** Country or region of birth (selected) of midwifery and nursing professionals by state or territory, 2011 (excluding those for whom country of birth is unknown)

	**Australian Capital Territory**	**New South Wales**	**Northern Territory**	**Queensland**	**South Australia**	**Tasmania**	**Victoria**	**Western Australia**
Australia	67.5%	66.0%	63.5%	68.3%	70.4%	81.7%	69.9%	51.7%
Total born overseas	32.5%	34.0%	36.5%	31.7%	29.6%	18.3%	30.1%	48.3%
United Kingdom and Ireland	6.7%	7.5%	7.5%	10.2%	12.3%	9.0%	7.0%	22.8%
Southeast Asia	5.6%	6.8%	5.0%	3.9%	3.7%	1.3%	6.5%	5.9%
Northeast Asia	3.1%	4.2%	0.7%	1.6%	2.8%	0.5%	2.6%	1.5%
South Asia	5.8%	3.5%	8.1%	2.5%	3.1%	1.0%	4.4%	3.2%
Sub-Saharan Africa	4.4%	2.6%	5.2%	2.5%	1.9%	1.6%	2.4%	6.2%
New Zealand	1.8%	2.4%	4.4%	5.9%	1.2%	1.7%	2.2%	4.1%
Other countries	5.1%	7.0%	5.8%	5.2%	4.5%	3.2%	5.1%	4.5%

The provision of census data enables an examination of the urban and rural health workforce by residence. The census defines “major urban” as towns and cities with populations of 100,000 people or more; the rest of the population has been classified as rural. Of the 70,151 medical practitioners for whom location was available, 58,337 (83.2%) individuals work in urban areas. Of the 238,953 nursing and midwifery professionals for whom location was available, 165,885 (69.4%) work in urban areas.

Of the medical practitioners working in rural locations, 49% are Australian-born compared with 46% of those working in urban areas. Doctors born in sub-Saharan Africa are overrepresented in rural locations (7% of those working in rural areas are born there compared with 4% of those working in urban areas). Conversely, 10% of the doctors working in urban areas were born in Southeast Asian compared with 5% of those working in rural areas.

As with medical practitioners, nursing and midwifery professionals working in rural areas are more likely to be Australia-born: 82% of those working in rural areas are Australia-born compared with 59% in urban areas. Only 1% of the nurses and midwives working in rural areas were born in South Asia or Southeast Asia.

To examine recent migration, an analysis was conducted on those medical practitioners and midwifery and nursing professionals who arrived in Australia between January 2001 and August 2011. Of the 14,268 medical practitioners who arrived in Australia between January 2001 and August 2011, 34.3% were born in South Asia, 14.2% in Southeast Asia, 13.5% in the United Kingdom and Ireland and 11.2% in sub-Saharan Africa.

Of the 31,478 midwifery and nursing professionals who arrived in Australia between January 2001 and August 2011, 23.3% were born in the United Kingdom and Ireland, 20.7% in South Asia, 15.2% in Southeast Asia and 13.8% in sub-Saharan Africa. From 2007 to 2011, 4,683 nurses and midwives moved to Australia from South Asia, representing 30.8% of all newly arrived nurses and midwives. This includes 3798 from India and 732 from Nepal.

Using the 2001 and 2006 data with the 2011 census data, the changing demographics of the Australian health workforce can be examined. Whereas 47.3% of the Australian medical practitioner workforce was Australian-born in 2011, the figure in 2006 was 51.9% (excluding the 1% for whom country of birth is unknown) and 57.1% in 2001. Similarly, Australian-born nurses and midwives declined from75.2% in 2001 to 69.8% in 2006 to 66.8% in 2011.

Overall, there were 15,168 more medical practitioners in Australia in 2011 than in 2006. This represents a 27.5% increase over the five years between the two censuses. This is to be taken in the perspective of an overall population increase in Australia of 8.3% between 2006 and 2011. Of those new medical practitioners, only 4716 (31.1%) were Australian-born.

**Figure 1:**
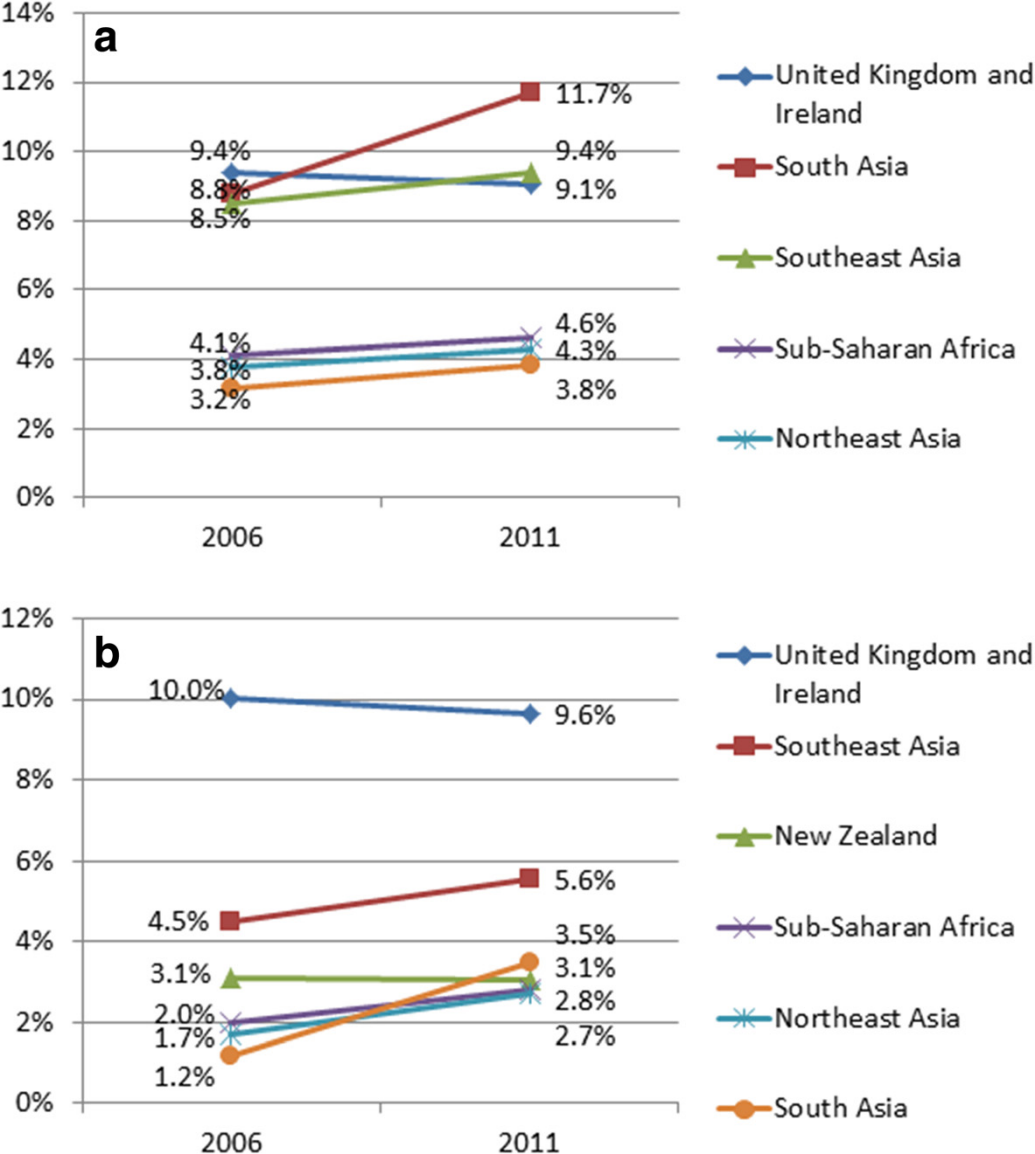
**Birthplace of health care workers in Australia. (a)** Birthplace of medical practitioners in Australia as a percentage of total in 2006 (*N* = 54,301) and 2011 (*N* = 69,666) (selected regions, excluding Australia). **(b)** Birthplace of nursing and midwifery professionals in Australia as a percentage of total in 2006 (*N* = 196,040) and 2011 (*N* = 236,403) (selected regions, excluding Australia).

Comparing 2006 data and 2011 data, there were 3397 more medical practitioners from South Asia (a 71.4% increase), including an increase of 61.5% in those from India (2807 to 4534) and a 352% increase from Nepal (23 to 104). As a percentage of total medical practitioners working in Australia for whom country of birth was known, the number of medical practitioners born in South Asia increased from 8.8% of total in 2006 to 11.7% in 2011 (Figure 1a). Increases were seen for those born in a number of other LMIC regions while the number of those born in the United Kingdom and Ireland decreased as a percentage of total number of medical practitioners.

Large increases in numbers of medical practitioners were also seen from Sri Lanka (76.7%) and from the Southeast Asia region, including the Philippines (65.5%) and a more than doubling of the number of medical practitioners born in Myanmar (from 186 to 394; 111.8%). In the five-year period, there were also 970 more individuals born in sub-Saharan African working as medical practitioners in Australia (43.3% increase) including a 53.2% increase in those from Zimbabwe, a 100% increase in those from Nigeria and a 208% increase in those from Botswana.

From 2006 to 2011, there were 38,903 more nurses and midwives working in Australia representing a 19.4% increase. An increase of 250%, or 5956 nurses, was seen in the numbers of nursing and midwifery staff born in South Asia. This included an increase in the number of Indian-born nurses from 1503 to 6200 (313% increase) and an increase in the number of Nepalese-born nurses from 144 to 1088 (656% increase). Increases of more than 80% were seen in the numbers of Indonesian- and Philippines-born nurses and midwives. There were also 2735 more nurses from sub-Saharan Africa working in Australia – an increase of 68.6%. This included a doubling of number from countries including Liberia, Nigeria, Sierra Leone, Ethiopia, Kenya and Zimbabwe.

The cost implications of health worker migration to Australia are considerable. A study by Mills and colleagues found that the average cost of medical education across eight African countries was US$28,535 [23]. Of the 6,708 medical practitioners who listed their year of arrival in Australia as 2007 to 2011, 4,777 (71.2%) were born in a LMIC. Assuming that all of those were trained overseas, then, extrapolating using the cost estimate from African countries, more than US$136 million was spent by LMIC governments and individuals over the past five years on medical education for medical practitioners who later moved to Australia. The same study, based on information provided by the Australian Medical Association, estimated that the cost of medical education for doctors in Australia is US$260,000. Therefore, for the 6,708 medical practitioners newly arrived in Australia over the past five years, we estimate that Australia and Australians have avoided US$1.7 billion in medical education costs that they would have otherwise had to spend to get the same number of health workers. Including nurses would invariably increase the savings considerably.

The issue of overseas-born health professionals working in Australia also needs to be looked at in the context of developing country workforce levels. Tables 4 and 5 examine, for selected countries, how doctor and nurse employment in Australia by those born in LMICs compares with workforce levels in those countries. For example, Sri Lanka only has 4.9 medical doctors per 10,000 population compared with 29.9 in Australia and the 2,058 Sri Lanka-born doctors in Australia represent 20% of the total number of doctors working in Sri Lanka, according to the WHO [24]. Similarly, while Nepal only has 4.6 nurses and midwives per 10,000 population (compared with 95.9 in Australia), the 1,088 Nepal-born nurses and midwives working in Australia (90% of whom arrived in Australia in the last ten years) represent more than 9% of the current nursing workforce in Nepal. Despite Australia’s relatively small population, those overseas-born health workers active in Australia represent considerable percentages of the domestic workforce in a number of LMICs with health worker shortages.

**Table 4:** Medical practitioner numbers in selected low- and middle-income countries compared with numbers in Australia

**Country**	**LMIC-born medical practitioners in Australia**	**LMIC-born medical practitioners who arrived in Australia between 2001 and 2011**	**Number of medical practitioners in LMICs**	**Number of medical practitioners in LMICs per 10,000 population (Australia: 29.9 per 10,000 population)**	**LMIC-born practitioners in Australia as a percentage of those in home country**
Fiji	313	108	380	4.5	82.4%
India	4,534	2,796	660,801	6.0	0.7%
Nepal	104	85	5,384	2.1	1.9%
Sri Lanka	2,058	977	10,279	4.9	20.0%
Tonga	15	7	30	2.9	50.0%
Zambia	76	28	649	0.6	11.7%
Zimbabwe	291	170	2,086	1.6	14.0%

Low- and middle-income country data from WHO (most recent year available) [24]. LMIC, low- and middle-income country.

**Table 5:** Midwifery and nursing professional numbers in selected low- and middle-income countries compared with numbers in Australia

**Country**	**LMIC-born midwifery and nursing professionals in Australia**	**LMIC-born midwifery and nursing professionals who arrived in Australia from 2001 to 2011**	**Number of midwifery and nursing professionals in LMICs**	**Number of midwifery and nursing professionals in LMICs per 10,000 population (Australia: 95.9 per 10,000 population)**	**LMIC-born professionals in Australia as a percentage of those in home country**
Fiji	1,733	409	1,660	19.8	104.4%
India	6,200	5,164	1,430,555	13.0	0.4%
Nepal	1,088	984	11,825	4.6	9.2%
Samoa	274	51	173	9.4	158.4%
Sierra Leone	130	118	991	1.7	13.1%
Sri Lanka	746	166	40,678	19.3	1.8%
Zimbabwe	2,053	1,766	9,357	7.2	21.9%

Low- and middle-income country data from WHO (most recent year available). LMIC, low- and middle-income country.

## Discussion

Analysis of the census data revealed that the number of foreign-born doctors, nurses and midwives as a percentage of the total has increased considerably between 2001 and 2011. More than half of all medical practitioners working in Australia are foreign-born, as are one third of nurses. The largest groups of foreign-born health workers come from South Asia, Southeast Asia, and the United Kingdom and Ireland. Western Australia has the highest rates of foreign-born health workers. A somewhat surprising finding was that there are in fact a higher percentage of Australia-born doctors and nurses working in rural and remote parts of Australia than there are in urban areas – despite efforts (including legislation) to encourage foreign-born doctors to go to rural areas. There has been a large recent influx of health workers from South Asia – India and Nepal in particular – with large increases from parts of sub-Saharan Africa as well.

The number of medical practitioners, nurses and midwives in the census data is lower than in the March 2012 Medical Board of Australia and Nursing and Midwifery Board data. The Medical Board [25] lists just over 91,000 medical practitioners compared with 70,231 from the census and the Nursing Board [26] data list more than 300,000 nurses compared with the approximately 240,000 in the census data. The Medical Board notes that more than 10% of registered medical practitioners do not spend most of their time working as such and therefore would not have been included in the census figures. The data also include more than 3,000 provisional practitioners who might not have been included in the census. The Nursing Board figures include enrolled nurses – excluded from the census – which, along with a number of nonpractising nurses, explains the difference between the two figures.

The DoHA data estimate that 39% of medical practitioners in Australia are foreign-trained [6] and census data suggest that just under 53% are foreign-born. This difference of approximately 14% might roughly represent those who were born overseas but trained in Australia.

The OECD data, which compare data across countries, are only available from 2000/1 (using 2001 Australian census data) but assert that, after New Zealand, Australia has the highest rates of foreign-born doctors and nurses in the OECD [18]. On average across the OECD, 11% of employed nurses and 18% of employed doctors were foreign-born in 2000/1. Hagopian and colleagues found that in the United States more than 23% of physicians received their medical training outside the USA, with more than 60% of those being trained in LMICs [27]. In the United Kingdom, one third of doctors qualified overseas [28]. The large number of Asian-born health workers found in this study is not unique to Australia; the OECD asserted that out of about 400,000 foreign-born doctors in 24 OECD countries, 32% were from Asia, while 25% of foreign-born nurses were from Asia [29].

The large number of overseas-born and overseas-trained health professionals potentially has an impact on medical education in Australia. The federal president of the Australian Medical Association recently lamented that locally trained junior doctors could not find internship places while Australia continues to import graduates from overseas [10]. Conversely, to increase internship places, more senior doctors capable of supervision are needed – a niche that overseas medical professionals could perhaps fill [6]. Taken together, health worker migration needs to be considered in the context of health workforce production sustainability in Australia [11].

The study also highlights the high number of health workers leaving LMICs to come to Australia – at least partly as a result of active recruitment. The case of the Philippines is remarkable. It is estimated that close to 15,000 nurses migrate overseas each year to 30 different countries, resulting in an estimated 30,000 unfilled nursing positions in the source country [1]. Similarly, a recent South African news report called for South Africa to import foreign health workers because more than 23,000 South African health professionals were working in high-income nations [30]. While there are considerable possible benefits to source countries in terms of remittances and return migration with enhanced skills, shortages of health workers have an immediate and direct impact on health service provision.

While country of qualification would certainly be a useful addition to the data presented here, to provide a robust picture of the health workforce and migration, the use of year of arrival to some degree mitigates the need for that data. Health workers who arrived in Australia within the past five years are more likely than not to be at least partly overseas trained due to the duration of training. The Australian Health Practitioner Regulation Agency, however, is in the process of developing a national health workforce dataset, which will hopefully include all such data [6], and Health Workforce Australia has included both country of birth and country of first qualification in its proposed national minimum dataset [31].

A further limitation is that the data presented here do not assess working hours but treat all those defined as health workers equally, irrespective of whether someone is working full-time or part-time. One Australian estimate stated that female doctors work 40% fewer lifetime hours than male doctors [32]. In addition, the census data are likely to underestimate the number of overseas-born health workers in Australia because there are likely to be some trained health workers who have migrated to Australia who no longer practise in the health field but work in education or in other fields and, therefore, were not captured by the self-defined occupation category of the census.

## Conclusions

This research highlights some of the challenges for Australia to achieve health worker sustainability. It also frames Australia’s health workforce realities in a global context – a necessity in our increasingly globalized world. In that context, as per the WHO and Commonwealth Codes of Practice, it should be asked whether Australia should only actively recruit from those countries that produce more health workers than are needed locally. Norway, for example, has begun implementing the code by scaling up medical education to ensure sustainability of its own health system and has formally stopped recruiting health workers from countries facing critical shortages [33]. Of the 24 high-income countries included in an OECD report, in 15 of them international medical graduates represent less than 20% of the doctor workforce, suggesting that other high-income countries have been able to address these challenges [18].

This is not an issue with a quick solution, as health worker education is a long-term process and demand currently overwhelms supply globally. But improved data collection and dissemination can hopefully assist in the forward planning needed for Australia to be able to meet domestic requirements while being a good global citizen and supporting its neighbours’ health condition.

## Abbreviations

AIHW: Australian Institute of Health and Welfare; DoHA: Department of Health and Ageing; LMIC: Low- and middle-income countries; OECD: Organisation for Economic Co-operation and Development; WHO: World Health Organization.

## Competing interests

The authors declare that they have no competing interests.

## Authors’ contributions

JN conceived the study, led the design and analysis and drafted the manuscript. AR assisted in analysis, contributed to the literature review and reviewed the manuscript. BC assisted in analysis and reviewed the manuscript. ALCM contributed to the design of the analysis and reviewed the manuscript. All authors read and approved the final manuscript.
